# MAP1981c, a Putative Nucleic Acid-Binding Protein, Produced by *Mycobacterium avium* subsp. *paratuberculosis*, Induces Maturation of Dendritic Cells and Th1-Polarization

**DOI:** 10.3389/fcimb.2018.00206

**Published:** 2018-06-21

**Authors:** Woo Sik Kim, Min-Kyoung Shin, Sung Jae Shin

**Affiliations:** ^1^Department of Microbiology and Institute for Immunology and Immunological Diseases, Brain Korea 21 PLUS Project for Medical Science, Yonsei University College of Medicine, Seoul, South Korea; ^2^Advanced Radiation Technology Institute, Korea Atomic Energy Research Institute, Jeongeup, South Korea; ^3^Department of Microbiology, Institute of Health Sciences, College of Medicine, Gyeongsang National University, Jinju, South Korea

**Keywords:** *Mycobacterium avium* subsp. *paratuberculosis*, MAP1981c, Crohn's disease, dendritic cells, Th1 immune response

## Abstract

*Mycobacterium avium* subsp. *paratuberculosis* (MAP) is the causative pathogen of chronic granulomatous enteropathy (Johne's disease) in animals, and has been focused on its association with various autoimmune diseases in humans, including Crohn's disease. The discovery of novel mycobacterial antigens and exploring their role in host immunity can contribute to the advancement of effective defense strategies including vaccines and diagnostic tools. In a preliminary study, we identified cellular extract proteins of MAP that strongly react with the blood of patients with Crohn's disease. In particular, MAP1981c, a putative nucleic acid-binding protein, showed high expression levels and strong reactivity to IgG and IgM in the sera of patients. Here, we investigated the immunological features of MAP1981c and focused on its interaction with dendritic cells (DCs), confirming its immunomodulatory ability. MAP1981c was shown to recognize Toll-like receptor (TLR) 4, and induce DC maturation and activation by increasing the expression of co-stimulatory (CD80 and CD86) and MHC class I/II molecules and the secretion of pro-inflammatory cytokines (IL-6, IL-1β, and TNF-α) in DCs. This DC activation by MAP1981c was mediated by downstream signaling of TLR4 via MyD88- and TRIF-, MAP kinase-, and NF-κB-dependent signaling pathways. In addition, MAP1981c-treated DCs activated naïve T cells and induced the differentiation of CD4^+^ and CD8^+^ T cells to express T-bet, IFN-γ, and/or IL-2, but not GATA-3 and IL-4, thus indicating that MAP1981c contributes to Th1-type immune responses both *in vitro* and *in vivo*. Taken together, these results suggest that MAP1981c is a novel immunocompetent antigen that induces DC maturation and a Th1-biased response upon DC activation, suggesting that MAP1981c can be an effective vaccine and diagnostic target.

## Introduction

*Mycobacterium avium* subsp. *paratuberculosis* (MAP) causes Johne's disease, which is a form of chronic gastroenteritis in ruminants (Motiwala et al., 2006). This disease is difficult to eradicate once it occurs in herds because of the shedding of MAP through feces and milk and the propagation of the infection during the latency period (Harris and Barletta, 2001). Recently, MAP has received increasing attention because it might cause zoonosis through infected food, such as meat and dairy products and several studies have reported a link between MAP infection and the pathogenesis of multiple human autoimmune diseases including type 1 diabetes mellitus, Hashimoto's thyroiditis, multiple sclerosis, sarcoidosis, Blau syndrome, and Crohn's disease (Quayle et al., 1992; Naser et al., 2004; Sechi et al., 2008; D'amore et al., 2010; Dow and Ellingson, 2010; Mameli et al., 2016). To date, the association of this pathogen with human diseases has been found by isolating MAP or detecting antibodies specific to MAP from the tissue samples of patients (Naser et al., 2000a,b, 2004; Sechi et al., 2005; D'amore et al., 2010; Dow and Ellingson, 2010; Cossu et al., 2011). Crohn's disease, which is a chronic inflammatory bowel disease in humans, is distinct from intestinal tuberculosis, though the clinical manifestations of the two diseases can be similar. When acid-fast staining bacilli were found in the intestine tract, *M. tuberculosis* or *M. bovis*, as agents of intestinal tuberculosis, were suspected; on the other hand, Crohn's disease is immunologically and pathologically different from intestinal tuberculosis, and therefore should be differentially diagnosed and treated (Sharma et al., 2016). Even though it has been suggested that MAP is a possible causative agent of Crohn's disease and/or other autoimmune diseases, this hypothesis has not been fully validated yet, mainly because of the slow growth and the difficulty in culturing this pathogen (Chamberlin et al., 2001).

MAP1981c, one of the cellular extract (CE) proteins isolated, is a protein with unknown function that showed a high expression level and strong immunoreactivity toward both IgG and IgM of serum from patients with Crohn's disease (unpublished data). In a study from Facciuolo and colleagues, MAP1981c induced a strong immune response in the Peyer's patch of the ileum, which is the main entrance site of MAP, and reacted with the serum of calves challenged with MAP (Facciuolo et al., 2016). Interestingly, we identified MAP1981c as a CE protein, while Facciuolo and colleagues reported it as a culture filtrate (CF) protein highly reactive to the blood of cattle with Johne's disease (Facciuolo et al., 2013). According to Facciuolo et al. the proteins identified by their MAP secretome analysis partially overlapped with MAP proteomes from cytoplasmic and membrane-enriched protein fractions identified in other studies. The authors suggested, therefore, that the methodologies used in cytoplasmic proteomes, cell wall proteomes, and secretomes allow the separation of unique MAP subproteomes with small overlaps. In addition, MAP1981c is an abundant protein that can be secreted or expressed in the CE fraction and is likely seroreactive for Johne's or Crohn's disease; therefore, it is worth studying this molecule as an immunological antigen.

MAP1981c is a hypothetical 245 amino acid (aa) protein with a C4-type zinc ribbon domain near the C-terminal region (aa 204–237) composed of two beta-strands acting as a support for two zinc knuckles, and two pairs of cysteines constituting two zinc knuckles located in a preserved sharp beta-turn, which is an arrangement that promotes the tetrahedral coordination of divalent Zn ions (http://pfam.xfam.org/family/zf-RING_7). Structural modeling has shown that this domain can bind to nucleic acids: therefore MAP1981c might be a nucleic acid-binding protein (Rigden, 2011). MAP1981c shows amino acid similarity with other predicted nucleic acid-binding proteins containing a Zn-ribbon from other mycobacteria such as Mav2210 (from *M. avium* 104, 99% similarity), MLM1938 (from *M. lepraemurium*, 96% similarity), Mb2254c (from *M. bovis* AF2122/97, 90% similarity), Rv2229c (from *M. tuberculosis* H37Rv, 90% similarity), and ML1638 (from *M. leprea* TN, 80% similarity), as well as with proteins from different bacterial species such as *Rhodococcus* sp. (67% similarity), and *Nocardiodes* sp. (66% similarity).

Although the function of MAP1981c is unknown, as a nucleic acid-binding protein it might be involved in DNA synthesis or replication. *Salmonella* is a gram-negative bacterium distinct from MAP; however, like MAP, this bacterium is also an intracellular pathogen and is exposed to the nitrosative stress in phagocytic cells (i.e., macrophages) where DNA replication is inhibited by DNA-binding Zn-metalloproteins involved in Zn mobilization (Schapiro et al., 2003). *Mycobacterium* is exposed to stressful environments, such as the hypoxic and acidic environment, nutrient deficiency, and oxidative stress when it infects the host; it is known to survive in a stressful environment through its slow replication and metabolism. Therefore, genes associated with these processes are thought to be related to the pathogenicity of mycobacteria (Via et al., 2008; Wang et al., 2015). In this study, we investigated MAP1981c, a putative nucleic acid-binding protein. We analyzed the immunological features of MAP1981c and focused on its potential interaction with dendritic cells (DCs). Our data indicate that this protein might be useful for vaccine target or early diagnosis of infection through its immunomodulation ability.

## Materials and methods

### Animals

Wild-type (WT), OT-I T-cell receptor transgenic (TCR), OT-II TCR transgenic, Toll-like receptor 2 (TLR2) knockout (TLR2 K/O), TLR4 K/O, myeloid differentiation primary response protein (MyD88) K/O, and Toll/IL-1R domain-containing adaptor inducing IFNβ (TRIF) K/O mice (All mice are C57BL/6 background) were purchased at 6–7 weeks of age from Jackson Laboratory (Bar Harbor, ME, USA). All animals were raised in a specific pathogen-free environment, and used in this study according to the regulations set forth by the Institutional Animal Care and Use Committee of the Yonsei University Health System (Permit number: 2015-0203).

### Reagents and antibodies

Recombinant mouse granulocyte-macrophage colony stimulating factor (GM-CSF) and interleukin (IL)-4 were purchased from JW CreaGene (Daegu, Korea). Lipopolysaccharide (LPS, from *Escherichia coli* O111:B4) were purchased from Invivogen (San Diego, CA, USA). The annexin V/propidium iodide (PI) kit was purchased from BD Bioscience (San Diego, CA, USA). ELISA kits for TNF-α, IL-6, IL-1β, IL-4, IL-12p70, and IL-10 were purchased from eBioscience (San Diego, CA, USA). The OT-I (OVA_257−264_) and OT-II (OVA_323−339_) peptides were synthesized by Abfrontier (Seoul, Korea). Fluorescein-conjugated antibodies (Abs) to, MHC-I, MHC-II, T-bet, and GATA-3 were purchased from eBioscience. Fluorescein-conjugated Abs to, CD11c, CD80, CD86, TNF-α, IL-12p70, IL-10, CD4, IFN-γ, IL-4, and CD8α were purchased from BD Bioscience. Anti-phosphorylated ERK1/2, -phosphorylated JNK, -phosphorylated p38, -phosphorylated IκB-a, IκB-a, NF-κB (p65), and lamin B were purchased from Santa Cruz Biotechnology (CA, USA). Anti-β-actin mAb (AC-15) and FITC-dextran (molecular mass, 40 kDa) were purchased from Sigma (St. Louis, MO, USA). All the pharmacological inhibitors were purchased from Calbiochem (San Diego, CA, USA).

### Expression and purification of MAP1981c protein

The MAP1981c gene was amplified from genomic DNA from MAP ATCC 19698 and the following primers: forward primer; the *Nde I* site is underlined, 5′-CGCCATATGAAAGCCGATGTAGCACAGCAG-3′ and reverse primer; the *Hind III* site is underlined, 5′-CCCAAGCTTCTGACCGGACCCCTTGACC-3′. The PCR-amplified full-length open reading frame (ORF) of MAP1981c gene was inserted into the plasmid pGEMT Easy Vector and sequences of MAP1981c gene in plasmid were confirmed. Next, the MAP1981c gene was cloned into the pET-22b (+) vector (Novagen, Madison, WI, USA) and then the clone was transformed into *E. coli* BL21 cells. The recombinant MAP1981c was expressed after induction with 1 mM isopropyl-β-D-thio-galactoside (IPTG) at 37°C for 6 h and then lysed and sonicated in lysis buffer supplemented with 20 mM Tris-HCl (pH 8.0), O,5 M Nacl, 20 mM imidazole, 1 mM dithiothreitol, and 1 mM phenylmethylsulfonyl fluoride. The recombinant proteins were purified by using an immobilized nickel-chelate (Ni-NTA) column (Invitrongen, Carlsbad, CA, USA) in accordance with the manufacturer's instruction. Endotoxin contamination was removed from the recombinant MAP1981c by incubation with polymyxin B (PMB)-agarose (Sigma) for 8 h at 4°C. Also, Residual LPS in the MAP1981c preparation was measured by an Limulus Amebocyte Lysate (LAL) assay (Lonza, Basel Switzerland) and was < 7 pg/mL (< 0.1 UE/mL) in MAP1981c preparations.

### Generation of bone marrow-derived dendritic cells (BMDCs)

Whole bone marrow cells from C57BL/6 mice were lysed of Red Blood Cells (RBCs) using RBC Lysing buffer (Sigma) and then cells were maintained in 6-well plates (1 × 10^6^ cells/mL; 2 mL/well) and cultured at 37°C in the presence of 5% CO_2_, using complete-RPMI 1640 (c-RPMI 1640) supplemented with 100 U/mL penicillin/streptomycin (Lonza, Basel, Switzerland), 10% fetal bovine serum (Lonza), GM-CSF (20 ng/mL) and IL-4 (0.5 ng/mL). Culture plates were added with 1 mL c-RPMI 1640 media per well on 3 and 6 days of culture. After 8 day culture, cells were harvested, stained with anti-CD11c mAb. Over 90% of the cells expressed CD11c.

### Annexin V and PI staining

BMDCs were treated for 24 h with LPS (100 ng/mL) or MAP1981c (1, 2, 5 μg/mL), respectably. After 24 h treatment, cells were harvested and stained with Annexin V and PI according to the manufacturer's instructions. Datas were collected on a FACSverse (BD Bioscience) and analyzed using FlowJo software (TreeStar).

### Analysis of cell surface molecules expression

Non-treated DCs (Con), LPS-treated DCs, and MAP1981c-treated DCs were first blocked with Fc Blocker (anti-CD16/32; ebioscience) for 20 min at room temperature and then stained with anti-CD11c, anti-CD80, anti-CD86, anti-MHC-I, and anti-MHC-II Abs for 20 min at room temperature. Expression of surface molecules in DCs was measured by flow cytometry and FlowJo software.

### Cytokine measurements

The cytokine concentrations were determined using ELISA kit following the manufacturer's instructions.

### LPS decontamination of recombinant MAP1981c through polymyxin B binding, boiling, and proteinase K treatment

For pretreatment with polymyxin B (PMB, Sigma), LPS and MAP1981c were incubated in a medium containing 10 μg/mL of PMB for 1 h at RT. For heat-denaturation, LPS or MAP1981c were incubated at 100°C for 1 h. For digestion of proteinase K (PK), LPS or MAP1981c were incubated along with 10 μg/mL soluble PK followed by heating for 20 min at 100°C to deactivate the enzyme, and subsequently added to DCs. After 24 h, cytokine levels in the supernatant of DCs were analyzed using ELISA.

### Analysis of antigen uptake ability

Non-treated DCs (Con), LPS-treated DCs and MAP1981c-treated DCs were equilibrated at 37or 4°C for 30 min and then cells treated with fluorescein-conjugated dextran (0.5 mg/mL). Cold PBS was added to stop the reaction. The cells were stained with anti-CD11c Ab and then CD11c^+^Dextran^+^ cells were measured by flow cytometry.

### Intracellular cytokine staining in DCs

On the 24 h after LPS or MAP1981c treatment in presence of GolgiPlug (BD Bioscience), cells were harvested and stained with anti-CD11c for 20 min at room temperature. The cells were fixed and permeabilized using Cyofix/Cytoperm kit (BD Bioscience) according to the manufacturer's instructions. Intracellular cytokine levels (TNF-α, IL-12p70, and IL-10) were detected with fluorescein-conjugated antibodies in a permeabilization buffer. Expression Levels of intracellular TNF-α, IL-12p70, and IL-10 in CD11c^+^ cells were analyzed by flow cytometry and FlowJo software.

### T cell proliferation assay

For T cells proliferation assay using OVA system, DCs were treated for 24 h with LPS or MAP1981c and then cells treated for 1 h with OT-I peptide (OVA_323−339_) and OT-II (OVA_257−264_). CD4^+^ and CD8^+^ T cells separated from OVA-specific TCR-transgenic mice were stained with 1 μM CFSE (with 5% FBS in RPMI, Invitrogen, Carlsbad, CA) for 10 min at 37°C water bath. Next, no OVA-treated and OVA-treated DCs (1 × 10^5^ cells/well) were co-cultured with CFSE-labeled CD4^+^ and CD8^+^ T cells (5 × 10^5^ cells/well) at an BMDC:T cell of 1:5. After 3 days co-culture, T cells were stained with anti-CD4 and CD8 Abs. CFSE^+^CD4^+^ and CFSE^+^CD8^+^ T cells were analyzed by flow cytometry and FlowJo software.

### Immunoblotting analyses

After stimulation with MAP1981c or LPS, cytosolic extracts from DCs were isolated using 100 μL lysis buffer supplemented with 50 mM Tris-HCl (pH 7.5), 150 mM NaCl, 1% Triton-X100, 1 mM EDTA, 50 mM NaF, 30 mM Na4PO7, 1 mM phenylmethanesulfonyl fluoride, 2 μg/mL aprotinin, and 1 mM pervanadate. Nuclear extracts from DCs were isolated using a Cellytic nuclear extraction kit (Sigma) according to the manufacturer's protocol. Immunoblotting was carried out as previously described (Kim et al., 2013). Target protein epitopes were labeled with primary Abs, and the results were visualized using an ECL Advance Western Blotting Detection kit (GE Healthcare, Little Chalfont, UK).

### Confocal laser scanning microscopy

DCs were incubated overnight on poly-L-lysine-coated glass coverslipt. After treatment with MAP1981c or LPS for 1 h, DCs were fixed in 4% paraformaldehyde, permeabilized in 0.1% Triton X-100, blocked with 2% BSA in PBS plus 0.1% Tween-20 (PBS/T) for 2 h, and incubated with 2% bovine serum albumin (BSA) in PBS/T containing anti-p65 mAb for 2 h at room temperature. The cells were incubated with an FITC-conjugated secondary Ab in a dark room for 1 h and stained with 1 μg/mL DAPI for 10 min at room temperature. The cell morphology and fluorescence intensity were observed by using a confocal laser scanning microscope (FV1000, Olympus, Japan).

### Analysis of conventional DC and T cell phenotype in spleens of MAP1981c-injected mice

The *in vivo* experiments for the activation of DC and T cells via MAP1981c injection were designed as described previously (Kim J. S. et al., 2015) and slightly modified. To analyze the activation of DCs in MAP1981c-injected mice, mice from the PBS and antigen groups were injected intravenously (IV) with PBS and 5 mg/kg MAP1981c. After 24 h of injection, splenocytes separated from PBS- or MAP1981c-injected mice were stained with the Live/Dead (L/D) cell staining kit (Invitrogen, Life Technologies, Naerum, Denmark), anti-CD11c, anti-CD8α, anti-CD80, anti-CD86, anti-MHC-I, and anti-MHC-II Abs. The expression of cell surface molecules in CD8α^+^CD11c^+^ and CD8α^−^CD11c^+^ conventional DCs (cDCs) was analyzed by flow cytometry. Next, to analyze the activation and polarization of T cells in MAP1981c-injected mice, mice from the PBS and antigen groups were injected IV with PBS and 5 mg/kg MAP1981c. Three days later, they were injected with PBS or 5 mg/kg MAP1981c again. Three days after the final injection, splenocytes were cultured in plates coated with Live/Dead, anti-CD3 mAb (0.5 μg/mL), and anti-CD28 mAb (0.5 μg/mL) in the presence of GolgiPlug. After 12 h, cells were harvested and stained with anti-CD3, anti-CD4, and anti-CD8 for 30 min at 4°C, and subsequently, cells were fixed and permeabilized using the Cyofix/Cytoperm kit. Following that, cells were stained with anti-IFN-γ and anti-IL-4 mAbs for 30 min at RT. Also, non-stimulated splenocytes were directly fixed and permeabilized using the Foxp3 Fixation/Permeabilization working solution (eBioscience) according to the manufacturer's instructions. Following that, cells were stained with anti-T-bet and anti-GATA-3 mAbs for 30 min at RT. The cytokines and transcription factors induced by T cells were analyzed by flow cytometry.

### Statistical analysis

Significant differences between samples were determined with Tukey's multiple comparison test and unpaired *t-*tests using statistical software (GraphPad Prism Software, version 5; GraphPad Software, San Diego, CA). The data in the graphs are expressed as the means. ^*^p < 0.05, ^**^p < 0.01, and ^***^p < 0.001 were considered statistically significant.

## Results

### MAP1981c is not cytotoxic to DCs

**Figure 1:**
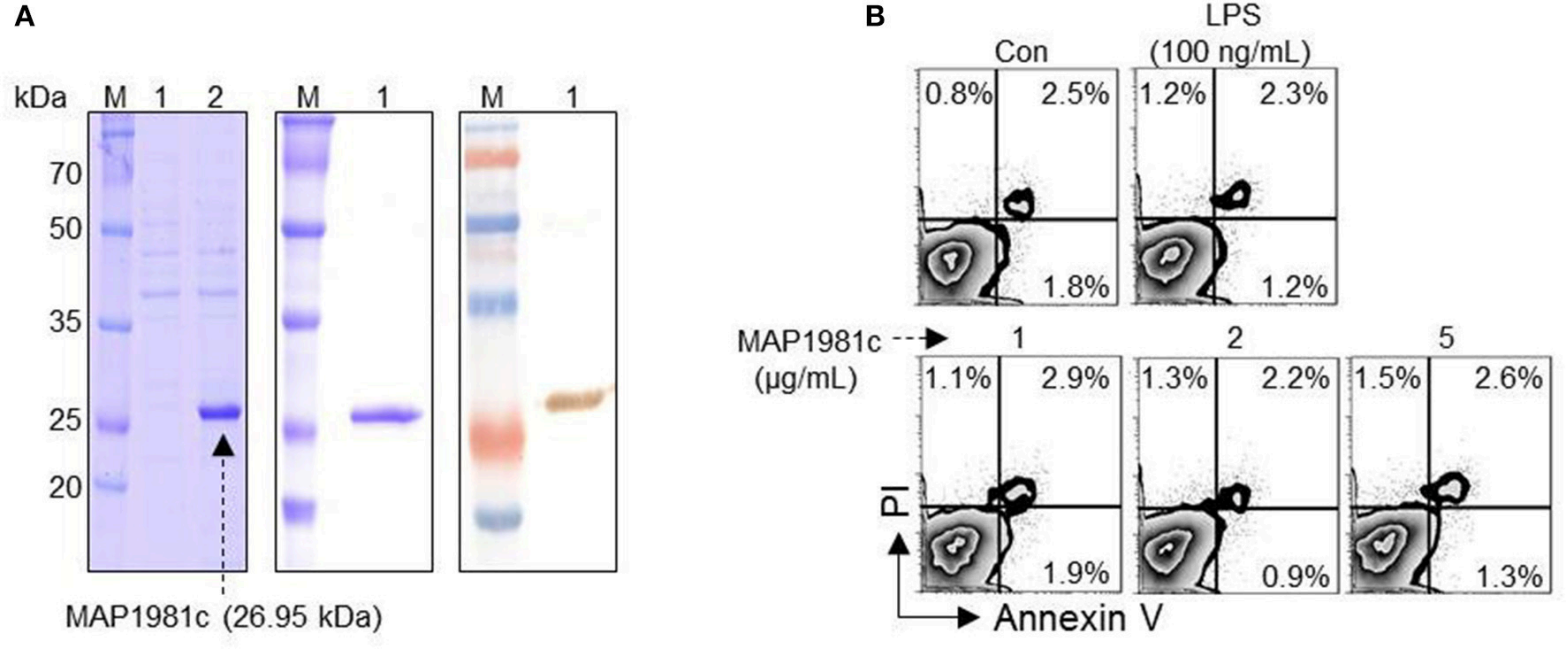
Cytotoxicity of recombinant MAP1981c protein. **(A)** Expression and purification of recombinant MAP1981c. (**A**, left panel) The recombinant MAP1981c was induced in *E. coli* by use of IPTG. Lane M, molecular weight marker; Lane 1, cell lysate before the addition of IPTG; Lane 2, cell lysate at 24 h after IPTG induction. (**A**, middle panel) The recombinant MAP1981c was purified by using Ni-NTA resin. The purified protein was subjected to SDS-PAGE. The gels were stained with Coomassie brilliant blue. (**A**, right panel) The purified protein was subjected to immunoblotting analysis with mouse anti-His Ab (1:1,000). **(B)** Cytotoxicity of the recombinant MAP1981c. The DCs were treated with MAP1981c (1, 2, or 5 μg/mL), LPS (100 ng/mL), or an equal volume of PBS as a control (Con). After 24 h of treatment, the cells were harvested and stained with annexin V and PI. The number in each quadrant indicated the percentage of positive cells. The results of one representative experiment out of three experiments producing similar results are shown.

The predicted molecular mass of MAP1981c is ~26.95 kDa. We confirmed this prediction by expressing recombinant MAP1981c followed by SDS-PAGE and western blotting assays (Figure 1A). Importantly, Annexin V/PI staining followed by flow cytometry analysis indicated that MAP1981c is not cytotoxic to DC, up to concentrations of 5 μg/mL MAP1981c (Figure 1B).

### MAP1981c induces phenotypic maturation of DCs and Th1-polarization

**Figure 2:**
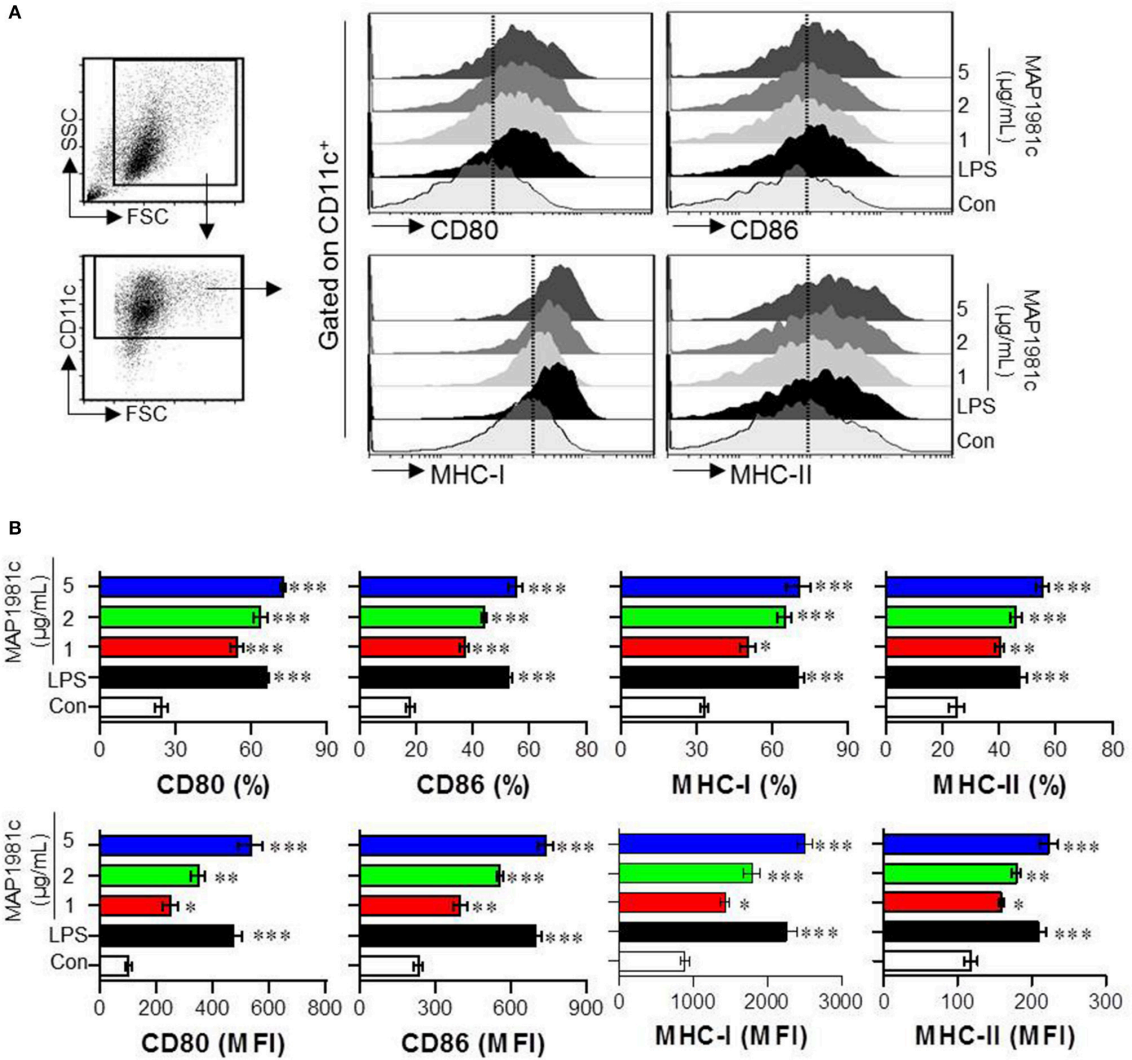
DC maturation induced by MAP1981c. The DCs were stimulated with MAP1981c (1, 2, or 5 μg/mL), LPS (100 ng/mL) or an equal volume of PBS as a control (Con) for 24 h. **(A)** Gating strategy for analysis of surface markers (CD80, CD86, MHC class I, and MHC class II) on stimulated CD11c+ DCs by using flow cytometry. **(B)** The percentages (top panel) and mean fluorescence intensities (MFI) (bottom panel) calculated from histograms are indicated as the mean ± SD of three independent experiments. *p < 0.05, **p < 0.01, ***p < 0.001 for the treatments compared with untreated DCs (Con).

Next, we investigated whether MAP1981c can induce the DCs maturation. Specifically, we analyzed the phenotypic maturation of DCs by analyzing the expression levels of co-stimulatory molecules (CD80 and CD86) and MHC class molecules (MHC-I and MHC-II) that are DC maturation markers, in the presence of MAP1981c (1, 2, or 5 μg/mL) or lipopolysaccharide (LPS, 100 ng/mL, used as a positive control). As shown in Figure 2, MAP1981c treatment significantly increased the expression of CD80, CD86, MHC-I, and MHC-II is a dose-dependent way.

**Figure 3:**
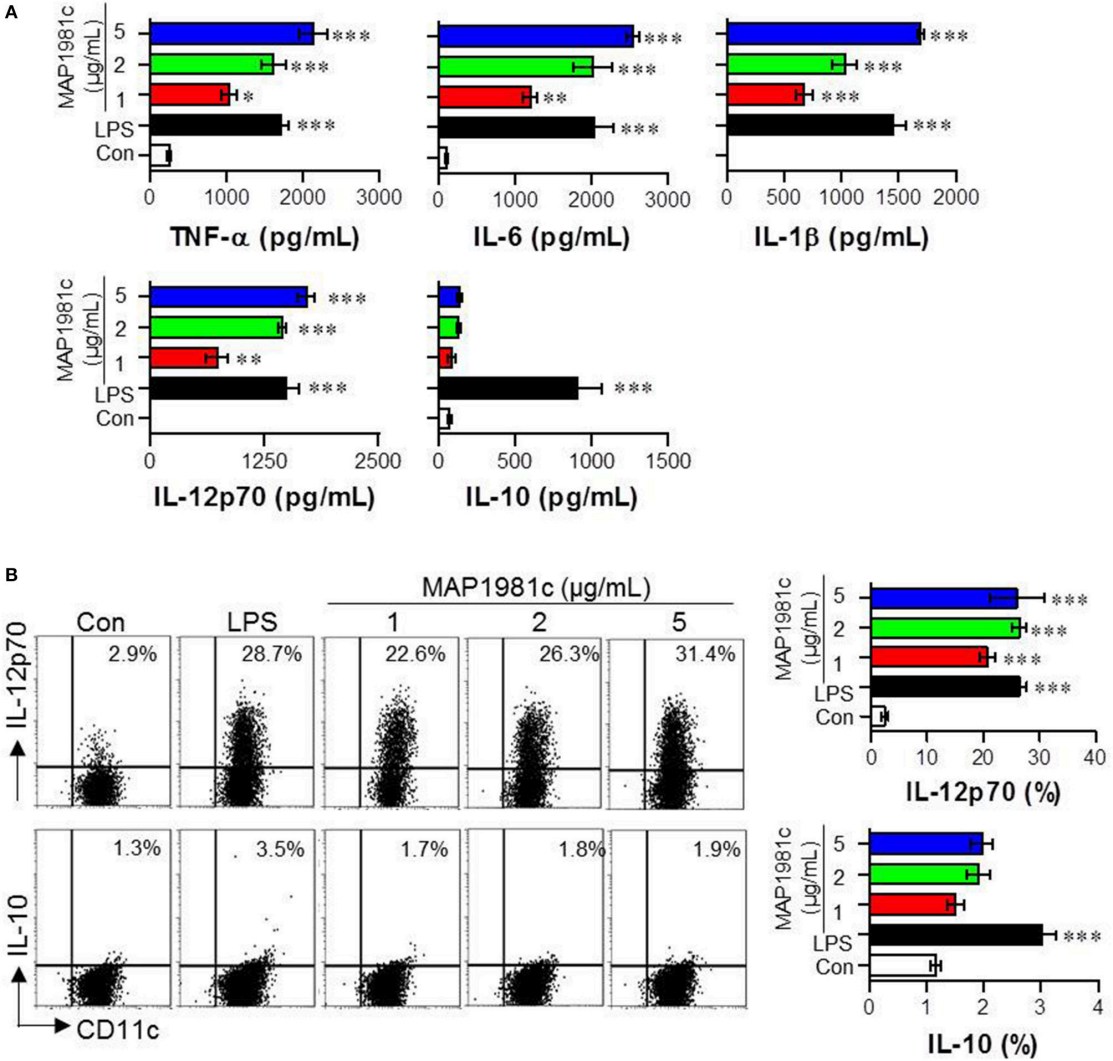
Cytokine production by MAP1981c stimulation in DCs. **(A)** The DCs were stimulated with MAP1981c (1, 2, or 5 μg/mL), LPS (100 ng/mL), or PBS (Con) for 24 h and the culture supernatants were analyzed for the amounts of TNF-α, IL-6, IL-1β, L-12p70, and IL-10 by using quantitative ELISA. **(B)** Dot plots of intracellular IL-12p70 and IL-10 in CD11c^+^ DCs. DCs were stimulated with MAP1981c (1, 2, or 5 μg/mL), LPS (100 ng/mL) for 12 h in the presence of GolgiPlug. The intracellular IL-12p70 and IL-10 levels were analyzed in CD11c^+^ DCs by flow cytometry. All bar graphs show the means ± SD of 3 samples. One representative plot out of three independent experiments is shown; *p < 0.05, **p < 0.01, ***p < 0.001 for the treatments compared with untreated DCs (Con).

**Figure 4:**
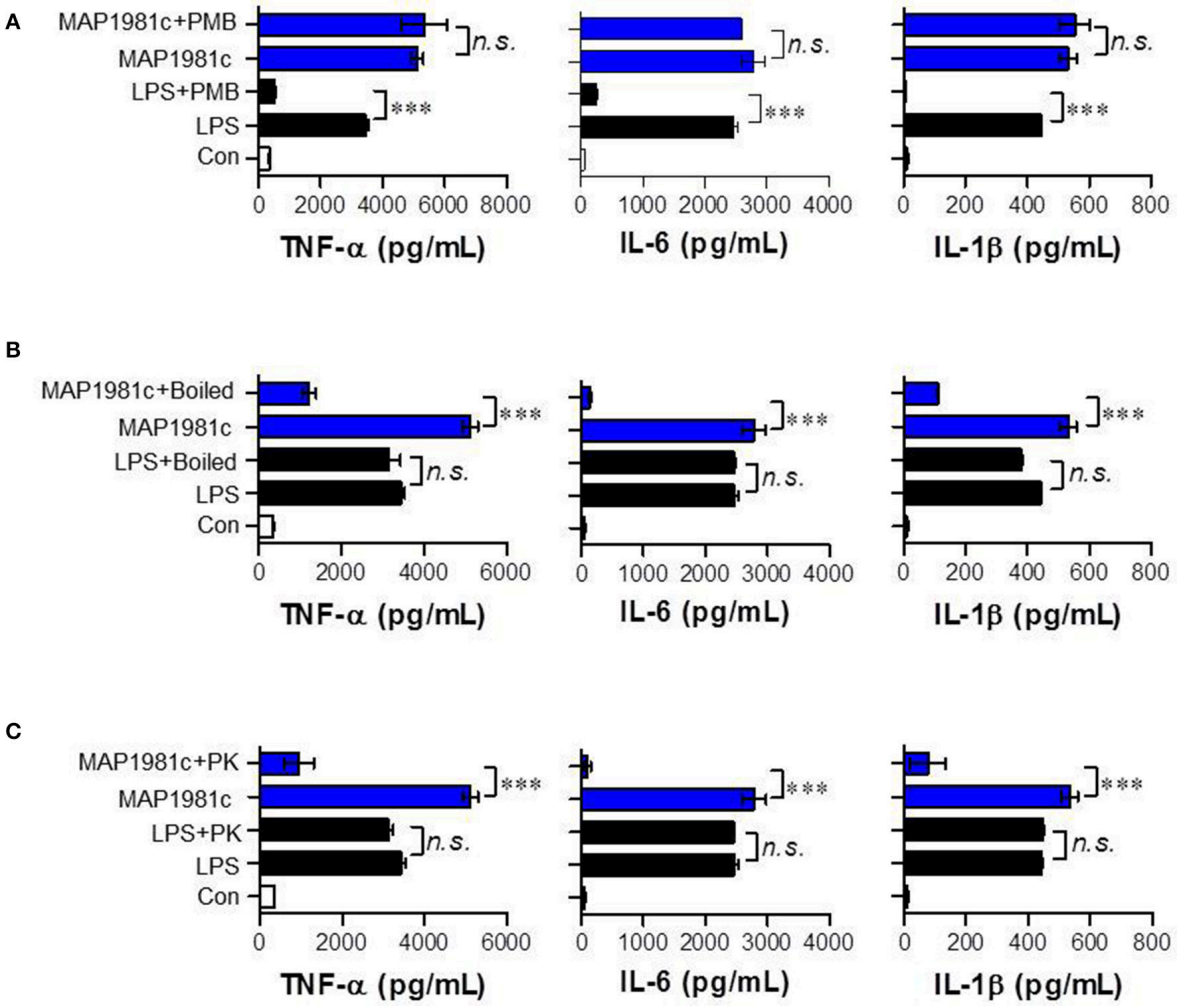
Confirmation of no LPS contamination of the recombinant MAP1981c. **(A)** DCs were treated with MAP1981c (5 μg/mL) and LPS (100 ng/mL) in presence and absence of PMB. **(B)** DCs were treated with MAP1981c, LPS, boiled MAP1981c, and boiled LPS. **(C)** DCs were treated with MAP1981c and LPS in presence and absence of PK. After 24 h treatment, the culture supernatants were analyzed for the amounts of TNF-α, IL-6, and IL-1β by using ELISA. All bar graphs show the means ± SD of 3 samples. One representative plot out of three independent experiments is shown. The value of n. s. was defined as having no significant effect. The value of ***p < 0.001 was considered to be statistically significant compared with MAP1981c-treated DCs or LPS-treated DCs.

The cytokines produced by mature DCs are potent signaling molecules. To determine the cytokines produced by MAP1981c-treated DCs, we next analyzed the expression of pro- (TNF-α, IL-6, and IL-1β) and anti-inflammatory (IL-10) cytokines, and that of a Th1 polarizing cytokine (IL-12p70). We found that MAP1981c strongly induced the expression of TNF-α, IL-6, IL-1β, and IL-12p70 in a dose-dependent manner, and did not affect the secretion of IL-10 (Figures 3A,B). We next confirmed the absence of endotoxin or LPS contamination of MAP1981c using the LAL assay (the endotoxin levels of MAP1981c are shown in the Materials and Methods section), heat denaturation, or treatment with PK or PMB. PMB treatment did not affect MAP1981c-induced cytokine production in DCs, whereas it significantly inhibited cytokine production in LPS-treated DCs (Figure 4A). Also, heat-denatured MAP1981c (Figure 4B) and co-treatment of DCs with MAP1981c and PK (Figure 4C) did not induce the secretion of TNF-α, IL-6, and IL-1β. Taken together, these results confirm that DC maturation is induced by MAP1981c and is not due to endotoxin contamination.

### MAP1981c treatment reduces dextran uptake by immature DCs

**Figure 5:**
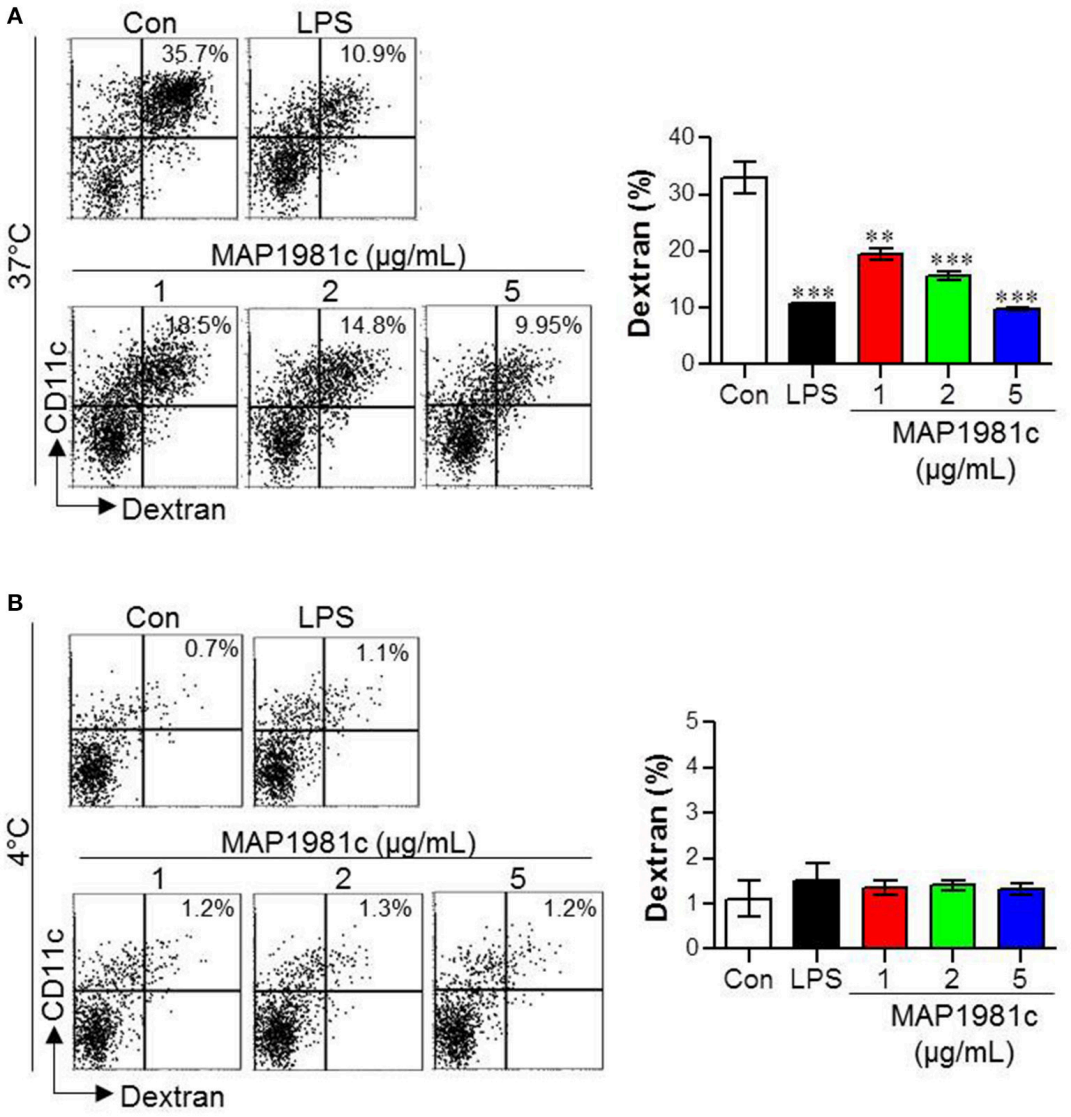
Endocytic activity induced by MAP1981c stimulation in DCs. The endocytic capacity was assessed at 37 **(A)** or 4°C **(B)** by the flow cytometric analysis of FITC-dextran uptake. The percentages of FITC-dextran-positive and CD11c^+^-positive cells are indicated. All bar graphs show the means ± SD of 3 samples. One representative plot out of three independent experiments is shown; **p < 0.01, ***p < 0.001 for the treatments compared with untreated DCs (Con).

DC maturation is characterized by antigen uptake ability: immature DCs exhibit high antigen uptake ability, while mature DCs exhibit low ability (Drutman and Trombetta, 2010). Therefore, we investigated antigen uptake ability during DC maturation induced by MAP1981c. Specifically, we evaluated the ability of DCs stimulated with various concentrations of MAP1981c (1, 2, 5 μg/mL) to uptake FITC-conjugated dextran at 37°C (Figure 5A), to detect positive cells, or 4°C (Figure 5B), to detect non-specific binding. Similar to the LPS-treated DCs, MAP1981c-treated DCs showed reduced FITC-dextran uptake ability compared with untreated DCs (Figure 5A). These results indicate that MAP1981c results in DC maturation and reduce antigen uptake.

### TLR4 and its downstream signaling is required for DC maturation induced by MAP1981c

**Figure 6:**
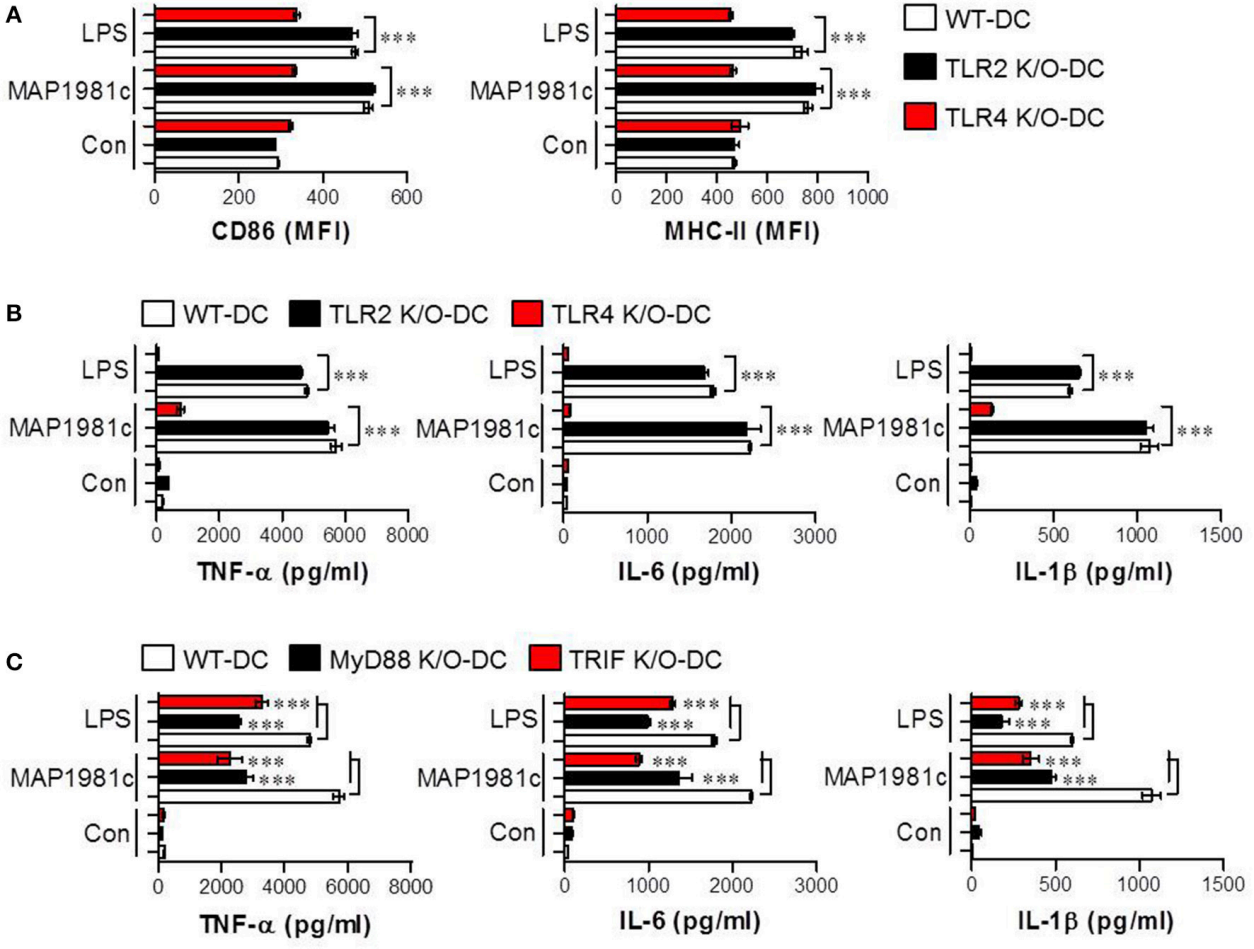
MAP1981c-induced DC maturation via TLR4 signaling. **(A,B)** WT-, TLR2 K/O, and TLR4 K/O-derived DCs were stimulated with MAP1981c (5 μg/mL) and LPS (100 ng/mL) for 24 h and analyzed for the expression of surface molecules (CD86 and MHC-II) and the production of pro-inflammatory cytokines (TNF-α, IL-6, and IL-1β) by flow cytometry **(A)** and ELISA **(B)**, respectively. **(C)** WT-, MyD88 K/O, and TRIF K/O-derived DCs were stimulated with MAP1981c (5 μg/mL) and LPS (100 ng/mL) for 24 h and analyzed for the production of TNF-α, IL-6, and IL-1β by ELISA. All bar graphs show the means ± SD of 3 samples. One representative plot out of three independent experiments is shown; ***p < 0.001.

Toll-like receptors (TLRs), such as TLR2 and TLR4, have been reported to recognize MAP in the context of murine macrophage and human PBMC activation (Ferwerda et al., 2007). Thus, to examine whether these receptors are involved in MAP1981c-induced DC maturation, the production of cytokines and the expression of surface molecules in WT-, TLR2 K/O-, and TLR4 K/O-derived DCs following treatment with MAP1981c were analyzed (Figure 6). We found that MAP1981c induced the expression of surface molecules (Figure 6A) and proinflammatory cytokine secretion (Figure 6B) in WT- and TLR2 K/O-derived DCs, but not in TLR4 K/O-derived DCs. Further, to confirm the relevance of TLR4 adaptor molecules, such as myeloid differentiation primary response protein (MyD88) and Toll/IL-1R domain-containing adaptor inducing IFNβ (TRIF) in MAP1981c-induced DC maturation, we analyzed the cytokine production in WT-, MyD88 K/O-, and TRIF K/O-derived DCs. As a result, the induction of cytokine production by MAP1981c treatment was significantly reduced in MyD88 K/O-, and TRIF K/O-derived DCs compared with that in WT-DCs (Figure 6C). These results indicate that TLR4/MyD88/TRIF-mediated signals are key players in MAP1981c-induced DC maturation.

### MAP1981c induces DC maturation through activation of the MAPKs and NF-kB signaling pathways

**Figure 7:**
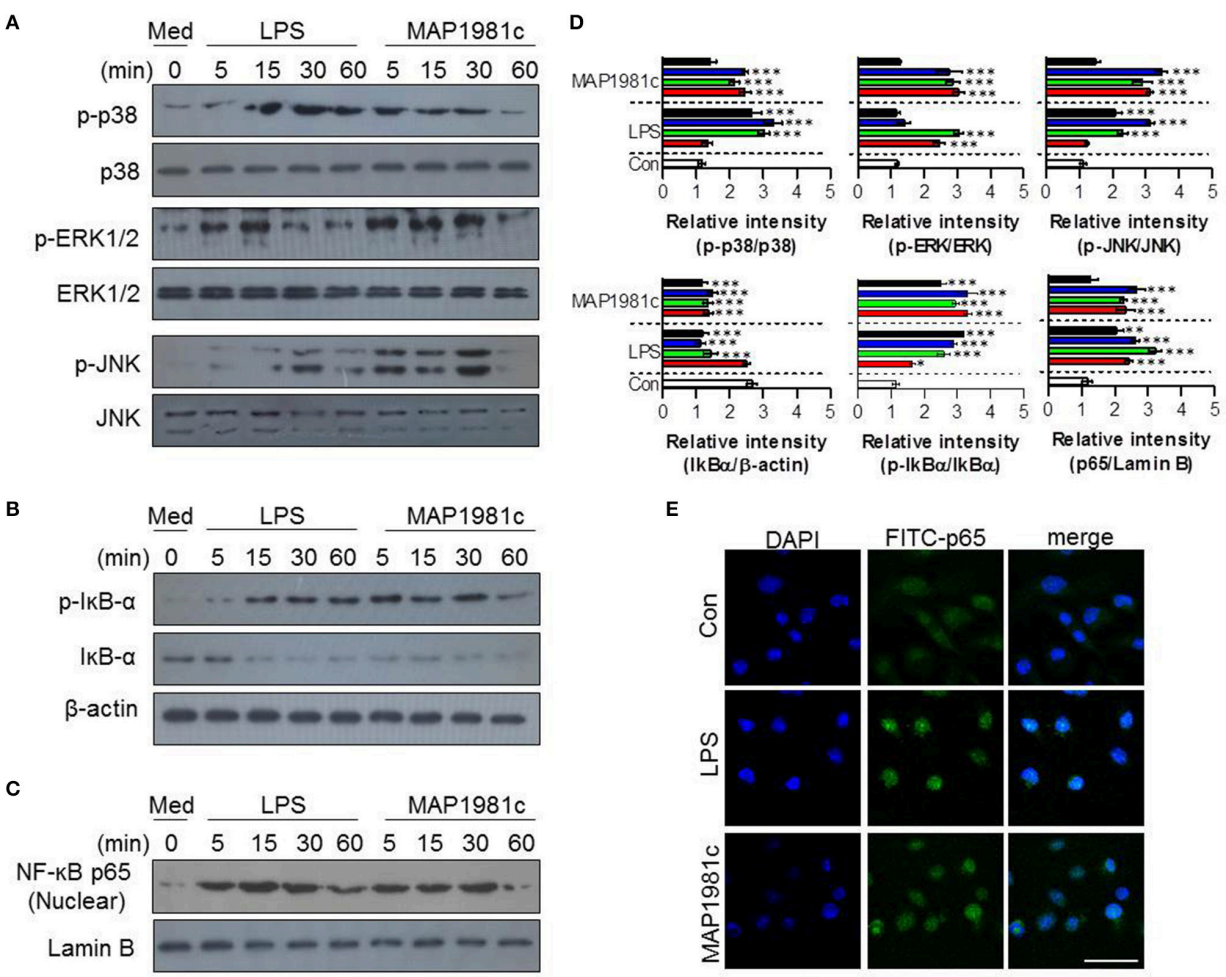
The activation of MAPK and NF-κB signal transduction induced by MAP1981c. DCs were stimulated with MAP1981c (5 μg/mL) for various time points (0, 15, 30, and 60 min) and then suspended in lysis buffer. Protein levels in cell lysate were evaluated by immunoblotting analysis. **(A)** Expressions of phospho-p38 (p-38), non-phospho-p38 (p38), p-ERK, ERK, p-JNK, and JNK in cell lysate. **(B)** Expressions of p-IkB-α, and IkB in cell lysate. β-actin; loading control for cytosolic fractions. **(C)** Expressions of NF-kB p65 in nuclear fractions. Lamin B; loading control for nuclear fractions. The images shown are representative of three independent experiments. **(D)** The relative band intensity of each protein is expressed as a percentage. The data are shown as means ± SD (*n* = 3 samples). ***p < 0.001 for the treatments compared with untreated DCs (0 min). **(E)** The effects of MAP1981c on the cellular localization of the p65 subunit of NF-κB in DCs. The images shown are representative of three independent experiments (scale bar = 5 μm).

**Figure 8:**
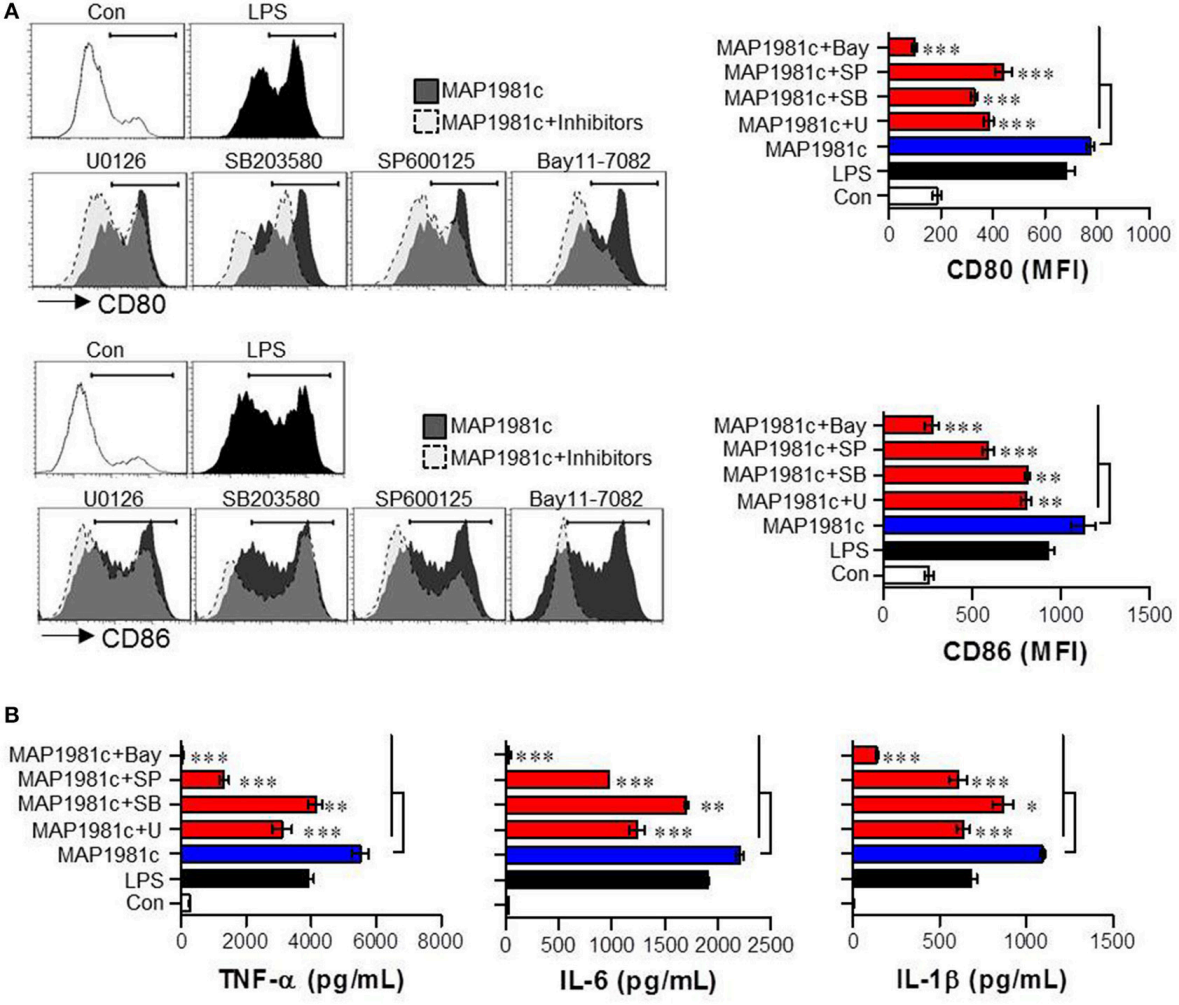
Inhibition of MAP1981c-induced DC maturation via pharmacological inhibitors of MAPKs and NF-kB signals. Following pre-treatment with pharmacological inhibitors (ERK; U0126, JNK; SP600125, p38; SB203580, NF-kB; Bay11-7082) for 1 h, cells were stimulated with MAP1981c (5 μg/mL) for 24 h. (**A**, left panel) Analysis of surface markers (CD80 and CD86) on stimulated CD11c^+^ DCs by using flow cytometry. The histograms shown are representative of three independent experiments. (**A**, right panel) The MFI calculated from histograms is indicated as the mean ± SD of three independent experiments. **p < 0.01, ***p < 0.001 for the treatments compared with MAP1981c-treated DCs (MAP1981c). **(B)** The culture supernatants were analyzed for the amounts of TNF-α, IL-6, and IL-1β by using ELISA. *p < 0.05, **p < 0.01, ***p < 0.001 for the treatments compared with MAP1981c-treated DCs (MAP1981c). U; U0126, SP; SP600125, SB; SB203580, Bay; Bay11-7082.

The mitogen-activated protein kinases (MAPKs) and nuclear factor kappa B (NF-kB) signaling pathways are essential for the expression and secretion of cytokines by mature DCs (Arrighi et al., 2001); however, the effect of MAP1981c on these pathways is unknown. We therefore examined whether MAP1981c could induce DC maturation via the MAPKs and NF-kB signaling pathways. We found that MAP1981c induced the phosphorylation of MAPKs (ERK, JNK, and p38; Figure 7A), phosphorylation and degradation of inhibitor of kB (IkB-α; Figure 7B), and the nuclear translocation of p65 (Figures 7C,E). Next, to confirm the role of these kinases in the DC maturation induced by MAP1981c, we analyzed surface molecule expression and cytokine production in DCs treated with MAPKs- and NF-kB-specific pharmacological inhibitors. Co-treatment with MAP1981c and these inhibitors significantly abolished the expression of surface molecules (CD80 and CD86; Figure 8A) and the production of pro-inflammatory cytokines (TNF-α, IL-6, and IL-1β; Figure 8B) compared to MAP1981c treatment. These results suggest that MAP1981c induces functional DC maturation via the MAPKs and NF-kB signaling pathways.

### MAP1981c-treated DCs promote naïve T cell proliferation and favor Th1 responses

**Figure 9:**
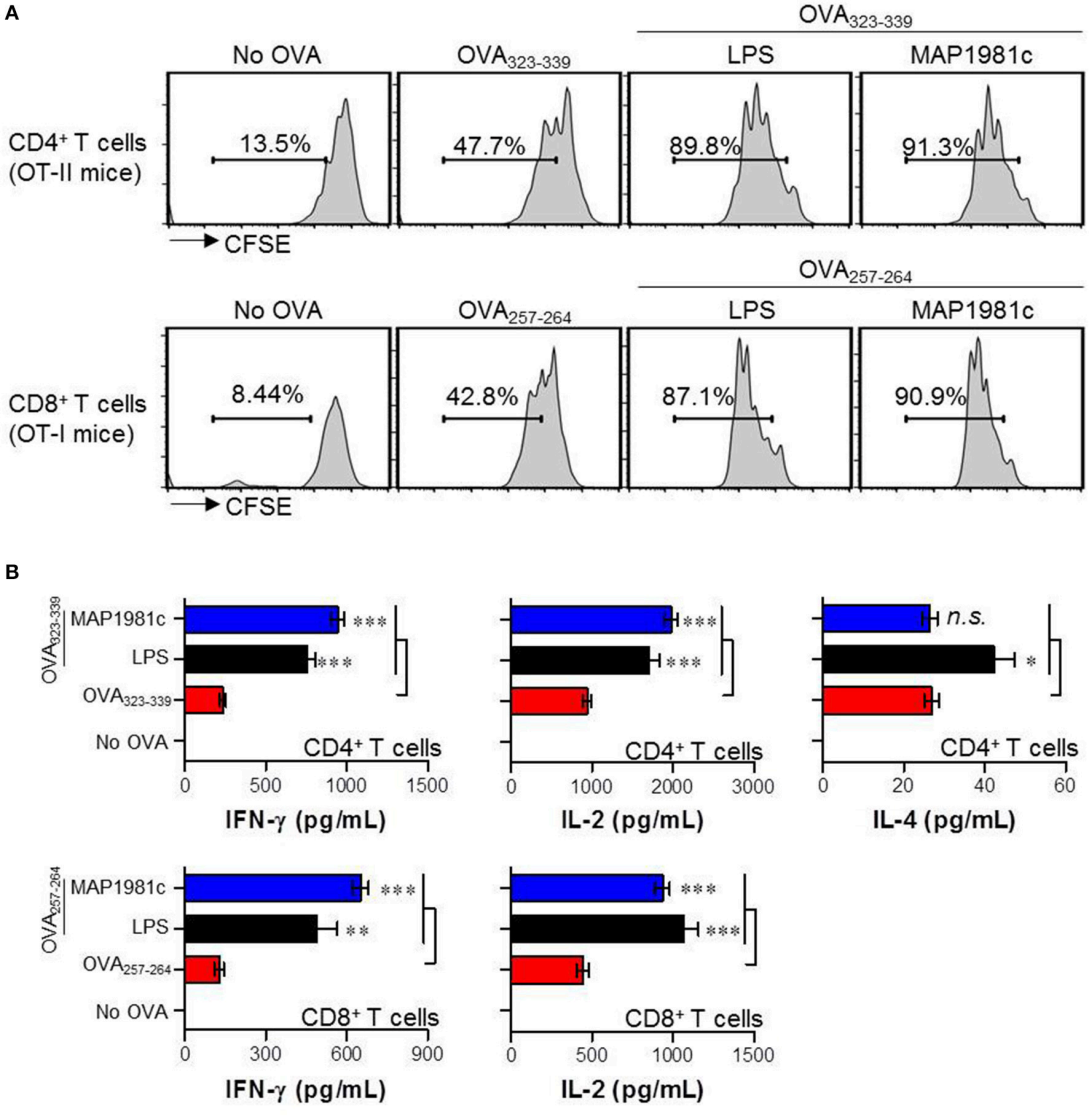
Proliferation and cytokine production of T cells induced by MAP1981c-treated DCs. **(A,B)** DCs were treated with MAP1981c (5 μg/mL) or LPS (100 ng/mL). After 24 h treatment, cells were stimulated with OVA_323−339_ or OVA_257−264_ of 1 μg/mL concentration for 1 h and then co-cultured with CFSE-labeled OVA-specific CD4^+^ and CD8 T cells. **(A)** After 3 days co-culture, cells were harvested and stained with anti-CD4 or CD8 Abs and then T cell proliferations were analyzed by flow cytometry. The histograms shown are representative of three independent experiments. **(B)** The culture supernatants were analyzed for the amounts of IFN-γ, IL-2, and IL-4 by using ELISA. All bar graphs show the means ± SD of 3 samples. One representative plot out of three independent experiments is shown; *p < 0.05, **p < 0.01, ***p < 0.001 for the treatments compared with OVA-treated DCs (OVA_323−339_-treated DCs or OVA_257−264_-treated DCs).

The primary role of DC is to interact with naïve T cells and induce T cell activation and differentiation (Lipscomb and Masten, 2002). Thus, we investigated the effect of DCs treated with MAP1981c on the proliferation and differentiation of OVA-specific CD4^+^ and CD8^+^ T cells using splenic T cells from OT-I and OT-II mice (splenic CD8^+^ T cells of OT-I mice expressing TCRs against OVA_257−264_ and splenic CD4^+^ T cells of OT-II mice expressing TCRs against OVA_323−339_). CD4^+^ and CD8^+^ T cells co-cultured with MAP1981c-treated DCs proliferated more than T cells co-cultured with non-treated DCs (Figure 9A). Additionally, we investigated the induction of major factors involved in the activation and differentiation of T cells exposed to MAP1981c-treated DCs. CD4^+^ T cells primed with MAP1981c-treated DCs produced high levels of IFN-γ and IL-2, important for polarization and activation of Th1-type cells; on the other hand, the expression of the Th2-type cytokine IL-4 was not affected (Figure 9B, top panel). Similarly, CD8^+^ T cells primed with MAP1981c-treated DCs produced high levels of IFN-γ and IL-2 cytokines (Figure 9B, bottom panel). These results suggest that MAP1981c-treated DCs can induce Th1 polarization in naïve CD4^+^ T cells, and activate naïve CD8^+^ T cells.

### MAP1981c induces the maturation of conventional DCs and promotes generation of Th1-type T cells *in vivo*

**Figure 10:**
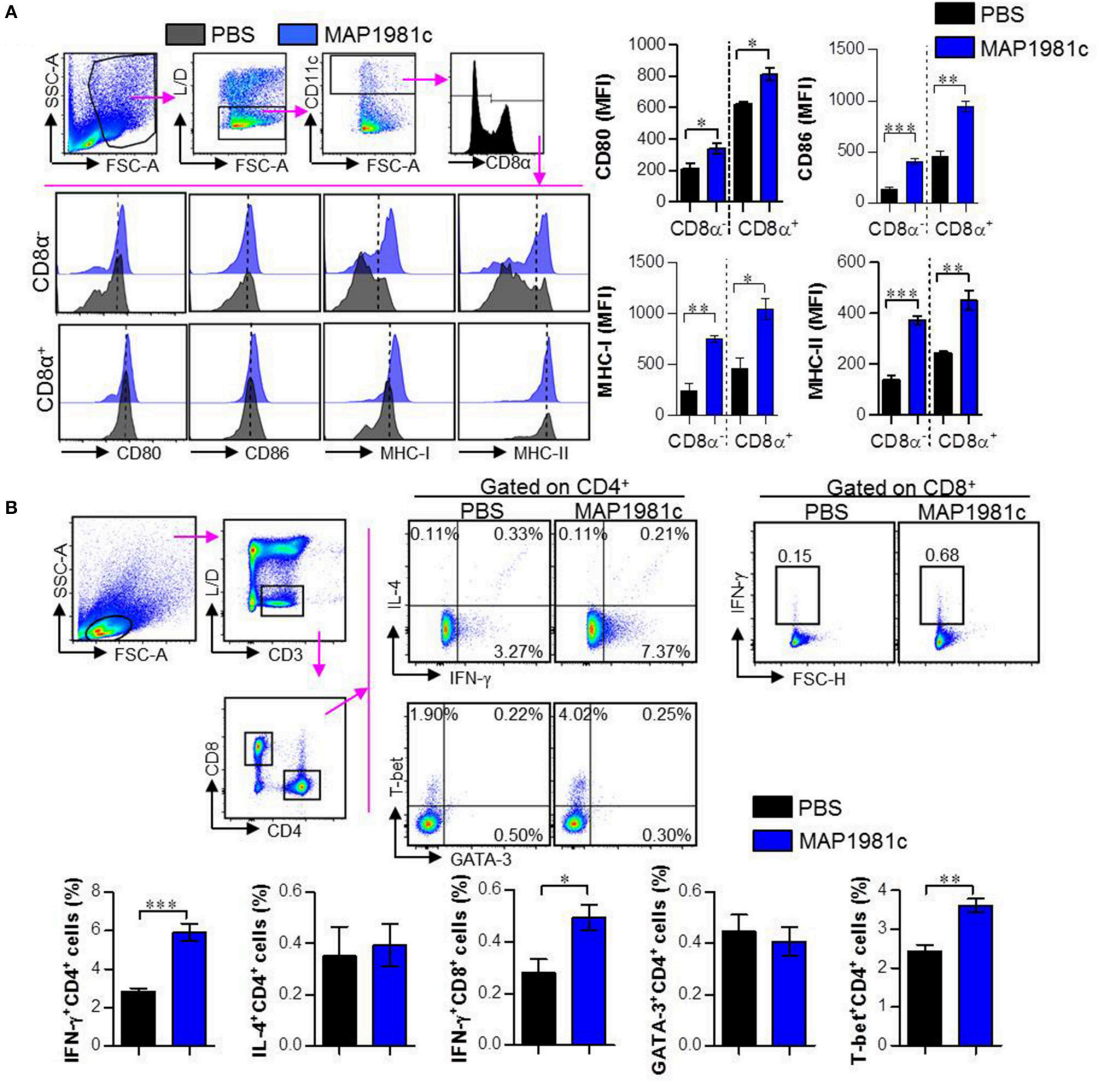
Induction of the splenic DC maturation and promotion of Th1-type cells by *in vivo* injection of MAP1981c. C57BL/6 mice (5 mice per group) were injected IV with 5 mg/kg MAP1981c or PBS and were analyzed 24 h later. **(A)** Single cells prepared from splenocytes of mice were stained with the Live/Dead (L/D) kit, anti-CD11c, anti-CD8α, anti-CD80, anti-CD86, anti-MHC-I, and anti-MHC-II Abs. The graphs show the expression levels of CD80, CD86, MHC-I, and MHC-II in CD8α^+^CD11c^+^ and CD8α^−^CD11c^+^ cells. All bar graphs show the means ± SEM from two independent experiments. **(B)** Mice (5 mice per group) of each group were injected with MAP1981c (5 mg/kg) or PBS, and after 3 days, were injected with MAP1981c (5 mg/kg) or PBS again. Single cells prepared from splenocytes of mice were stimulated with anti-CD3/CD38 Abs in the presence and absence of GolgiPlug for 12 h, and then the cells were stained L/D kit, anti-CD3, anti-CD4, anti-IFN-γ, anti-IL-4, anti-T-bet, and anti-GATA-3 Abs and analyzed by flow cytometry. The graphs show the expression levels of IFN-γ, IL-4, GATA-3, and T-bet in CD3^+^CD4^+^ or CD3^+^CD8^+^ T cells. All bar graphs show the means ± SEM from two independent experiments. *n.s*, No significant effects, *p < 0.05, **p < 0.01, ***p < 0.001.

Based on the above *in vitro* results, we evaluated whether MAP1981c can induce DC maturation, generation of Th1-type T cells, and activation of T cells *in vivo* (Figure 10). The *in vivo* experiment was performed as described in the Materials and Methods section. MAP1981c-injected groups showed increased surface molecule expression in splenic CD8α^+^ cDCs (CD8α^+^CD11c^+^) and CD8α^−^ cDCs (CD8α^−^CD11c^+^) compared with PBS-injected groups, indicating DC maturation (Figure 10A). We also observed that the frequencies of Th1-type T cells (IFN-γ^−^producing CD4^+^ T cells and T-bet-expressing CD4^+^ T cells) and activated CD8 T cells (IFN-γ^−^producing CD8^+^ T cells) were generally increased in the MAP1981c-injected groups compared with the PBS-injected groups. However, no significant differences between PBS- and MAP1981c-injected groups were observed for the frequency of Th2-type T cells (IL-4^−^producing CD4^+^ T cells and GATA-expressing CD4^+^ T cells; Figure 10B).

## Discussion

*Mycobacterium avium* subsp. *paratuberculosis* (MAP) is the cause of gastroenteritis in ruminants and is linked to the pathology of Crohn's disease in humans. In the present study, we characterized a putative DNA binding protein, MAP1981c, isolated from cellular extracts of MAP human isolates, which is reactive against the sera of patients with Crohn's disease. We found that MAP1981c contributes to the development of T cell-mediated immunity through DC activation. MAP1981c recognizes TLR4, and promotes DC maturation and activation via MyD88- and TRIF-dependent pathways, followed by the activation of MAP kinases and NF- κB. In addition, MAP1981c-treated DCs activated naïve T cells and induced the differentiation of CD4^+^ and CD8^+^ T cells to express T-bet, IL-2, and IFN-γ, but not GATA-3 and IL-4, thus inducing Th1-type immune responses both *in vitro* and *in vivo*.

DCs are special antigen presenting cells that can lead to immune responses by engaging both innate and adaptive immunity, and are necessary for T cell activation and differentiation (Lipscomb and Masten, 2002; Kim T. H. et al., 2015). Therefore, it is important to study the mechanism through which MAP antigen induces DC maturation as well as the immune responses induced during MAP pathogenesis. MAP-infected macrophages or DCs travel to the draining lymph nodes, and the interaction of migrated cells with T cells is important for an appropriate immune response: the T cell-mediated immune response can induce infected macrophages to eliminate intracellular bacteria or kill infected macrophages (Koets et al., 2015). The progression of mycobacterial infection has been proposed to be linked to the Th1/Th2 shift in the host (Magombedze et al., 2014). The infected animals initially exhibit a cell-mediated CD4^+^ T cell response to MAP, characterized by the production of interferon-γ (Th1 response); however, as the disease progresses, the Th1 response diminishes while the antibody response to MAP (Th2 response) increases. Therefore, the conversion from Th1 to Th2 response occurs simultaneously with disease progression and bacterial excretion (Magombedze et al., 2014). Interestingly, Th1-inducible mycobacterial antigens may be useful as vaccine and diagnostic antigens during the early infectious stages, since the Th1/IFN-γ response has been demonstrated as a key factor in controlling mycobacterial infection (Moliva et al., 2017).

Recently, several MAP antigens have been studied because of their immunological role in the DC-mediated immune responses. A study has found that a MAP fibronectin attachment protein (ModD) and MAP1305 induce Th1-type responses by activating DCs, and the response is induced through toll-like receptor 4 (TLR4) (Lee et al., 2009, 2014). Ag85, CobT (35-kDa protein), and malate dehydrogenase, all produced by MAP, are T cell immune modulating antigens leading to T cell proliferation and IFN-γ production (Rosseels et al., 2006; Byun et al., 2012; Kim et al., 2018). Additionally, the 70 kDa heat shock protein of MAP can induce DC maturation and activation through the NF-κB and MAPK signaling, confirming the induction of protective immune responses to MAP through T cell polarization (Hoek et al., 2010). On the contrary, Basler et al. showed inhibition of DC maturation and induction of a tolerogenic DC phenotype with lowered expression of MHCII, CD86, and TNF-α when DCs were treated with supernatants from MAP-infected macrophages. However, when DCs were infected with MAP, the same authors confirmed induction of DC maturation, followed by decreased IL-12 and increased IL-10 production (Basler et al., 2013). In this respect, MAP could induce DC maturation as well as tolerogenic immune responses. Nevertheless, several MAP antigens, capable of induction of DC maturation and Th1 immune responses, may be potentially useful for vaccine production or as diagnostic tools.

In the present study, recombinant MAP1981c was produced and analyzed for its immunological role on DCs. DCs are involved in the initiation of the immunopathological reaction; after the migration of activated DCs to the lymphoid organs, the DCs ultimately induce antigen-specific T cell responses (Choi et al., 2015). The results of the present study showed that MAP1981c induced activation and maturation of DCs, manifested by the increased expression of cell surface markers such as co-stimulatory signals (CD80, CD86), MHC class I and II and pro-inflammatory cytokines including TNF-α, IL-6, IL-1β, and IL-12p70, and decreased antigen uptake capacity (Figures 2, 3, 5). TLR2 and TLR4 play an important role in the initial recognition of mycobacterial antigens in macrophages and DCs, which are known to affect innate and acquired immunity (Carmona et al., 2013). Recently, it has been reported that a TLR4 agonist may play an important role as an immunomodulator in the treatment of cancer (Jung et al., 2011). Moreover, the study has shown that signals from MyD88- and TRIF-dependent pathways are particularly strong immunological stimuli (Jung et al., 2011). MAP1981c interacted with TLR4 and induced the activation of MyD88- and TRIF-dependent pathways and MAPK and NF-κB signaling pathways (Figures 6, 7), which are associated with the maturation of DCs and the expression of proinflammatory cytokines (Rescigno et al., 1998; Kim J. S. et al., 2015). DC activation by MAP1981c induced naïve CD4^+^/CD8^+^ T cell proliferation and prompted CD4^+^ and CD8^+^ T cells to express T-bet, IL-2, and IFN-γ, but not GATA-3 and IL-4, thus inducing Th1-type immune responses both *in vitro* and *in vivo* (Figures 9, 10).

As indicated above, we decided to focus on MAP1981c in this study because of its strong immune response in both patients with Crohn's disease and cattle with Johne's disease. Similarly, in calves challenged with MAP, Facciuolo et al. found a stronger antibody reactivity for MAP1981c 2 months post infection (PI) than 1 month PI and pre-infection (Facciuolo et al., 2016). Here, we demonstrated that MAP1981c can induce DC maturation and activation and a Th1-type immune response. Consequently, our study shows that MAP1981c could be a novel immunocompetent antigen that induces Th1 cell polarization through its interaction with DCs, suggesting that MAP1981c may be a new target for MAP vaccine production and MAP infection diagnosis.

## Author contributions

WK performed the majority of the experiments. WK, M-KS, and SS conceived the study, analyzed all the data, and wrote the manuscript. M-KS and SS critically revised the manuscript. All the authors discussed the results and commented on the manuscript.

### Conflict of interest statement

The authors declare that the research was conducted in the absence of any commercial or financial relationships that could be construed as a potential conflict of interest.
